# The Anatomy of the Future

**Published:** 1890-02

**Authors:** 


### SELECTIONS.

### The Anatomy of the Future.

Some years ago an elderly professor of anatomy expressed his thankfulness that in the branch he taught there was no harassing progress. Physiology and chemistry, he said, were undergoing changes from one day to another, but anatomy was always anatomy, a bone was always a bone, and there was no wearisome struggle to keep up with the rushing progress of the times. And such is the view of anatomy entertained by many of the profession. Not a few of us think, when we look back over a number of years spent in active practice, that we started on our journey with much more baggage than was necessary in the way of memorized anatomical truths; that we could have done with a smaller quantity, but that that smaller quantity should have been better selected. We question whether the anatomy taught in our colleges to-day is the anatomy of the future. Is it not rather a result of the labor of successive generations of book-writers, each showing more eagerness to add new facts to the mass than to examine into and verify the old ones?

The anatomy of the future will not resemble that taught now. The scope of the subject requires enlarging, and for the narrowing memory work done to-day a broad view of the whole must be substituted. In other words, the student should first acquire a knowledge of general and comparative anatomy, and subsequently study its application to the human body. To begin work with the descriptive anatomy of man is working backward. Such a course has been likened to the study of an ultimate twig of a tree by a person who is ignorant of the character of the larger branches, of the trunk, and of the soil on which the tree grew.

Embryology urgently demands attention as a necessary introduction to the study of the parts of the body and as a subject the knowledge of which is of direct practical advantage in daily professional life. Observe how important has become the anatomy of the embryo in connection with the study of disease. The disposition of the layers of the embryo must be clearly understood to enable one to understand the structures of the body and the diseases developing in those structures. Studies such as these lighten the student's task. Take, for instance, the arrangement of the great vessels in the root of the neck. The disposition of these structures is learned commonly by the study of a scheme or diagram, perhaps by the friendly aid of a cunningly devised "tip." The relations of these vessels could, by the study of development, be so impressed upon the student's mind as never to be forgotten. Can any of our readers forget what a bugbear the peritoneum was? Can you put your hands on your hearts and say that you thoroughly understand all about it now? And yet, when the the peritoneum is studied from its early and simple state there will be no longer any difficulty either in comprehending or remembering the arrangement of its folds.

The very great advance made of late years in the study of nervous diseases renders a change in anatomical teaching very necessary. In our day the anatomy of the arteries was paramount, and the nervous system occupied a position of secondary importance. Yet the nerves, their cutaneous distribution, their communications with central ganglia and with one another, are matters of daily, almost hourly, consideration in the round of a doctor's visits, while of the arteries, over which we spent such time and such labor, there is rarely occasion to think. The practitioner is constantly meeting with nervous manifestations the correct interpretation of which renders a clear knowledge of nerve distribution necessary, while it is possible for him to practice for the whole course of his natural existence without ever being called upon to tie an artery. Should such a demand be made, he will seize the bleeding point in the wound, secure it, save his patient, and then go home to study the vascular system afterward. The statement has been made, we think by the late

Dr. Fothergill, that the chances of an ordinary practitioner being called upon to tie the subclavian are almost equal to those of his meeting his death by lightning stroke.

To render the study of anatomy attractive and at the same time thoroughly useful, a complete change in or present system is called for, and teachers will soon have to consider the importance of comparative anatomy and of embryology as introductory studies, and the necessity of putting the nervous system in its proper place as the most important department of the human body, and not waste their energies in teaching the relations of arteries with which the student is unlikely ever to have anything to do.—*Editorial in N. Y Med. Journal.*

